# Lithium-Acetate-Mediated Biginelli One-Pot Multicomponent Synthesis under Solvent-Free Conditions and Cytotoxic Activity against the Human Lung Cancer Cell Line A549 and Breast Cancer Cell Line MCF7

**DOI:** 10.1100/2012/109432

**Published:** 2012-04-19

**Authors:** Harshita Sachdeva, Diksha Dwivedi

**Affiliations:** Department of Chemistry, Faculty of Engineering and Technology, Mody Institute of Technology and Science, Lakshmangarh 332311, Rajasthan, India

## Abstract

Various Biginelli compounds (dihydropyrimidinones) have been synthesized efficiently and in high yields under mild, solvent-free, and eco-friendly conditions in a one-pot reaction of 1,3-dicarbonyl compounds, aldehydes, and urea/thiourea/acetyl thiourea using lithium-acetate as a novel catalyst without the addition of any proton source. Comparative catalytic efficiency of lithium-acetate and polyphosphoric acid to catalyze Biginelli condensation is also studied under neat conditions. The reaction is carried out in the absence of any solvent and represents an improvement of the classical Biginelli protocol and an advantage in comparison with FeCl_3_ *·*6H_2_O, NiCl_2_ *·*6H_2_O and CoCl_2_ *·*6H_2_O that were used with HCl as a cocatalyst. Compared to classical Biginelli reaction conditions, the present method has advantages of good yields, short reaction times, and experimental simplicity. The obtained products have been identified by spectral (^1^H NMR and IR) data and their melting points. The prepared compounds are evaluated for anticancer activity against two human cancer cell lines (lung cancer cell line A549 and breast cancer cell line MCF7).

## 1. Introduction

Multicomponent reactions (MCRs) have emerged as an efficient and powerful tool in modern synthetic organic chemistry because the synthesis of complex organic molecules from simple and readily available substrates can be achieved in a very fast and efficient manner without the isolation of any intermediate [1, 2]. Therefore, the discovery for new MCRs and improving the already-known MCRs are of considerable interest. Perusal of literature revealed that the Biginelli reaction, which was discovered more than a century ago, is one of the most important MCRs for the synthesis of dihydropyrimidinones based on acid-catalyzed three component condensation of *β*-dicarbonyl compound, an aldehyde, and urea or thiourea [3, 4]. 

 The development of new strategies for the preparation of complex molecules in neat conditions is a challenging area of organic synthesis. The pioneering work of Toda et al. [5] has shown that many exothermic reactions can be accomplished in high yield by just grinding solids together using mortar and pestle, a technique known as “grindstone chemistry,” which is one of the “green chemistry techniques.” Reactions are initiated by grinding, with the transfer of very small amounts of energy through friction [6]. In addition to being energy efficient, grindstone chemistry also results in high reactivity and less waste products. Such reactions are simple to handle, reduce pollution, comparatively cheaper to operate, and may be regarded as more economical and ecologically favorable procedure in chemistry [7]. Generally, solid-state reactions occur more efficiently and more selectively than does the solution reaction, since molecules in the crystals are arranged tightly and regularly [8].

 The classical Biginelli reactions were conducted under strongly acidic conditions, which suffer from poor yields, and long reaction time and sensitive functional groups are lost during the reaction conditions. This has lead to the development of several new methodologies, which improve the yields compared to original procedure. These new strategies involve the combinations of Lewis acids and/or transition metal salts, for example, BF_3_·OEt_2_, montmorillonite (KSF), polyphosphate esters, and reagents like InCl_3_ [9], LiBr [10], TMSCl/NaI [11], LaCl_3_·7H_2_O [12], CeCl_3_·7H_2_O [13], Mn(OAc)_3_·2H_2_O [14], InBr [15], FeCl_3_ and HCl [16], ytterbium triflate [17], Iodine [18], ZnCl_2_ [19], CoCl_2_ [20], and so forth. Many Lewis acids and transition metal salts have been found to catalyze this reaction, but they still have limitations like high cost, limited availability, prolonged reaction duration, and the use of strong acids. The combination of solvents and long reaction time, costly chemicals/catalysts, makes this method environmentally hazardous. Therefore, development of simple, efficient, clean, high yielding, and environmentally friendly approaches using new catalysts for the synthesis of these compounds is an important task of organic chemists.

 Although the original Biginelli protocol of acid-catalyzed condensation in ethanol is effective method but still various modifications of solvents and catalyst are the subject of this synthesis. During our quest to develop novel catalysts for multicomponent reactions, we have found that lithium-acetate is effective promoter of Biginelli reaction. Literature survey revealed that so far some metal acetates [21] seem to have been utilized to catalyze Biginelli reactions in the presence of solvents but lithium-acetate has not been utilized so far. 

**Scheme 1:**
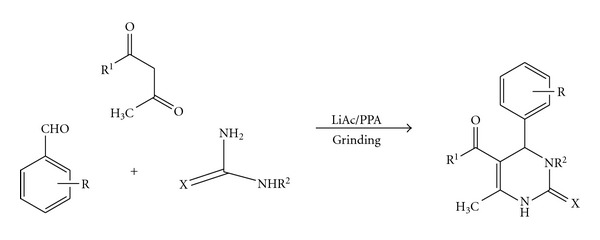


As per our ongoing efforts to synthesize privileged class of compounds [22, 23] and our interest in Lewis acid applications for the Biginelli reaction, herein we wish to report for the first time a novel, simple, and efficient methodology for the synthesis of 3, 4-dihydropyrimidin-2(1H)-ones and thiones (DHPMs) by the reaction of aldehydes, 1,3-dicarbonyl compounds, and urea/thiourea using catalytic amount of lithium-acetate and (polyphosphoric acid) PPA under solvent-free conditions (Scheme 1). Further, comparative catalytic efficiency of lithium-acetate and PPA to catalyze Biginelli condensation is also studied under neat conditions.

## 2. Results and Discussion

The current method not only preserves the “one-pot” protocol of Biginelli condensation but also favors environmentally benign reaction conditions of saving energy consumption. Literature survey revealed that there is little report [24] on its use in the synthesis of DHPMs and this technique is superior to the existing methods, since grinding does not require solvents leading to safe and environmental friendly synthesis. Furthermore, the proposed technique does not require external heating or cooling at any stage, leading to energy efficient synthesis providing high yields of products.

In order to optimize the reaction conditions, the synthesis of compound 1 was used as a model reaction and a mixture of benzaldehyde, ethylacetoacetate, and urea in the presence of lithium-acetate/PPA was magnetically stirred at room temperature in different solvents (Table 1).

 The best yield (73%) was obtained when 0.5 equivalents of lithium-acetate/PPA (entries 3 and 6), 1 equivalents of both benzaldehyde and ethylacetoacetate, and 2 equivalents of urea were magnetically stirred in acetonitrile (CH_3_CN)/Tetrahydrofuran (THF), respectively, for 7-8 hours.

It seems that THF is a much better solvent in PPA catalyzed reaction (entry 3) whereas CH_3_CN is better in lithium-acetate-promoted reaction in terms of yields and time than all other tested solvents (entry 6). However, under solvent-free conditions reaction was fast and 90–95% yield of DHPMs was obtained in 10–15 minutes (Table 2). 

Encouraged by these results, and due to increasing demand in modern organic processes of avoiding expensive purification techniques and large amount of solvents, we examined the reactivity of our catalysts under solvent-free conditions. The three component cyclocondensation reaction was carried out either by stirring a mixture of aromatic aldehyde (1 eq.), 1,3-dicarbonyl compounds (1 eq.), urea/thiourea/acetyl thiourea (2 eq.), and catalytic amount of lithium-acetate/PPA (0.5 eq.) for 30–35 minutes at 70–80°C temperature (Method I) or by grinding the reactants together for 5–10 minutes at room temperature (Method II). After the completion of reaction (as monitored by TLC), the reaction mixture was cooled to room temperature and poured onto crushed ice, filtered, and recrystallized by using either ethanol or ethyl acetate and pet ether (1 : 1) to afford pure product. As reaction was carried out under solvent-free conditions, clean products are obtained. It has been observed that yield obtained by Method I is 10–15% better than that obtained in case of Method II.

 With the aim of improving the reaction yields, we attempted to add different equivalents of catalyst to the reaction mixture and the best condition to prepare DHPMs was achieved when reaction was carried out in the presence of 0.5 eq. of catalyst. The use of large amount of catalyst (1 eq, 1.5 eq., or 2 eq.) does not increase the yields. To check promoter efficiency of catalyst and reproducibility of the reaction, different aldehydes were reacted with urea/thiourea/acetyl thiourea to give 27 different compounds. It was found that catalysts employed (PPA/Lithium-acetate) differed in their efficiency in terms of yields and purity.

 In all cases studied, the three-component reaction proceeded smoothly to give the corresponding DHPMs (1–27) in satisfactory yields. Most importantly, aromatic aldehydes carrying either electron-donating or electron-withdrawing substituents reacted very well to give the corresponding DHPMs with high purity in moderate-to-good yields. Another important feature of this procedure is the tolerance of various functional groups, such as methoxy, halides, and hydroxy, to the reaction conditions. Thiourea/acetyl thiourea (entries 2, 8, 13, 15, 19, and 24–27) has been used with similar success to provide corresponding S-dihydropyrimidinone analogues, which are also of interest due to their biological activities. It should be noted that PPA or lithium-acetate was used as the sole promoter agent in neutral media and reaction proceeded without using any additional proton source while for others previously reported [16, 20] hydrates of metal halides such as Fe (III), Ni (II), and Co (II), a catalytic amount of conc. HCl was needed as a Bronsted acid cocatalyst. Melting points of most of synthesized DHPMs were found to be much closer to reported DHPMs indicating high purity of the compounds (Table 2). The structure of all the dihydropyrimidinones prepared is characterized by IR and ^1^H NMR and are well correlated with the available literature data.

### 2.1. Evaluation of Anticancer Activity

The synthesized compounds 6, 16, 18, 23, 24 were evaluated [31] for anticancer activity against human lung cancer cell line A549 and 2, 3, 7, 12, 22 against human breast cancer cell line MCF7. The cell lines were grown in RPMI 1640 medium containing 10% fetal bovine serum and 2 mM L-glutamine. For present screening experiment, cells were inoculated into 96 well microtiter plates in 90 *μ*L at plating densities, depending on the doubling time of individual cell lines. After cell inoculation, the microtiter plates were incubated at 37°C, 5% CO_2_, 95% air, and 100% relative humidity for 24 h prior to addition of experimental drugs. After 24 h, one plate of each cell line was fixed *in situ* with TCA, to represent a measurement of the cell population for each cell line at the time of drug addition (**T** **z**). Experimental drugs were solubilized in appropriate solvent at 400-fold the desired final maximum test concentration and stored frozen prior to use. At the time of drug addition, an aliquot of frozen concentrate was thawed and diluted to 10 times the desired final maximum test concentration with complete medium containing test article at a concentration of 10^−3^. Additional three, 10-fold serial dilutions were made to provide a total of four drug concentrations plus control. Aliquots of 10 *μ*L of these different drug dilutions were added to the appropriate microtiter wells already containing 90 *μ*L of medium, resulting in the required final drug concentrations.

### 2.2. Endpoint Measurement

After compound addition, plates were incubated at standard conditions for 48 hours and assay was terminated by the addition of cold TCA. Cells were fixed *in situ* by the gentle addition of 50 *μ*L of cold 30% (w/v) TCA (final concentration, 10% TCA) and incubated for 60 minutes at 4°C. The supernatant was discarded; the plates were washed five times with tap water and airdried. Sulforhodamine B (SRB) solution (50 *μ*L) at 0.4% (w/v) in 1% acetic acid was added to each of the wells, and plates were incubated for 20 minutes at room temperature. After staining, unbound dye was recovered and the residual dye was removed by washing five times with 1% acetic acid. The plates were air-dried. Bound stain was subsequently eluted with 10 mM trizma base, and the absorbance was read on an ELISA plate reader at a wavelength of 540 nm with 690 nm reference wavelengths. Percent growth was calculated on a plate-by-plate basis for test wells relative to control wells. 

*Percent Growth was expressed as the ratio of average absorbance of the test well to the average absorbance of the control wells ∗ 100.*

Using the six-absorbance measurements time zero (*Tz*), control growth (*C*), and test growth in the presence of drug at the four concentration levels (*Ti*), the percentage growth was calculated at each of the drug concentration levels. 

*Percentage growth inhibition was calculated as*

(1) $$\begin{array}{cc} & \left[\frac{\left(Ti-Tz\right)}{\left(C-Tz\right)}\right]\times 100\;\text{for}\;\text{concentrations}\;\text{for}\;\text{which}\;Ti\\ & \;\;\geq Tz\;\left(Ti-Tz\right)\;\text{positive}\;\text{or}\;\text{zero},\\ & \left[\frac{\left(Ti-Tz\right)}{Tz}\right]\times 100\;\text{for}\;\text{concentrations}\;\text{for}\;\text{which}\;Ti\\ & \;\;<Tz.\;\left(Ti-Tz\right)\;\text{negative}. \end{array}$$

The dose response parameters were calculated for each test article. Growth inhibition of 50% (GI50) was calculated from [(*Ti* − *Tz*)/(*C* − *Tz*)] × 100 = 50, which is the drug concentration resulting in a 50% reduction in the net protein increase (as measured by SRB staining) in control cells during the drug incubation. The drug concentration resulting in total growth inhibition (TGI) was calculated from *Ti* = *Tz*. The LC50 (concentration of drug resulting in a 50% reduction in the measured protein at the end of the drug treatment as compared to that at the beginning) indicating a net loss of cells following treatment is calculated from

(2) $$\;\left[\frac{\left(Ti-Tz\right)}{Tz}\right]\times 100=-50.\;$$

Values were calculated for each of these three parameters if the level of activity was reached; however, if the effect was not reached or was exceeded, the values for that parameter were expressed as greater or less than the maximum or minimum concentration tested. 

**Figure 1:**
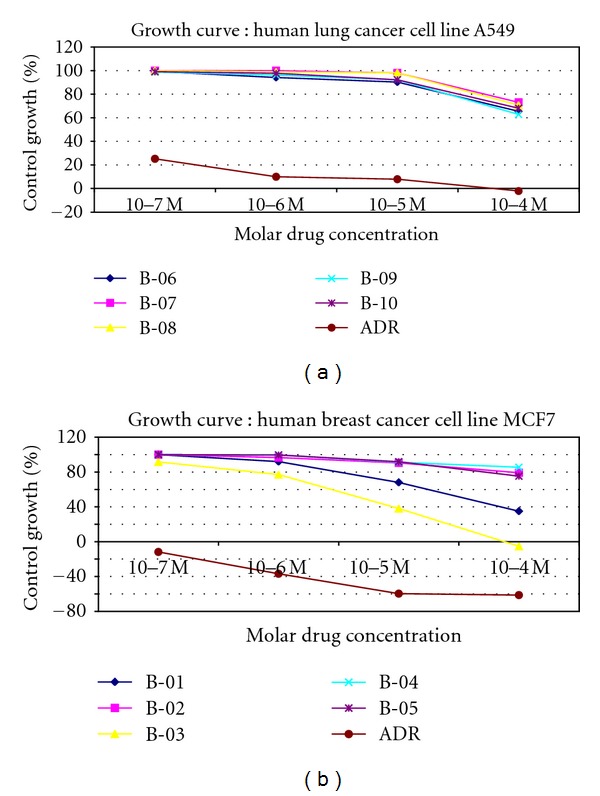
Invitro cytotoxic effects of DHPM's against two Human Cancer Cell Lines.

 The data of anticancer activity in terms of % control growth at different molar drug concentration are shown in Tables 3 and 4. Unfortunately, all the evaluated compounds showed poor activity against human cancer cell lines. Growth curves are presented in Figure 1.

**Definition 2.2 s**

LC50: concentration of drug causing 50% cell kill; GI50: concentration of drug causing 50% inhibition of cell growth; TGI: concentration of drug causing total inhibition of cell growth; ADR: adriamycin, positive control compound. GI50 value of ≤10^−6^ molar (i.e., 1 *μ*molar) or ≤ 10 *μ*g/mL is considered to demonstrate activity in case of pure compounds. For extracts, GI50 value ≤ 20 *μ*g/mL is considered to demonstrate activity Italic test values under GI50 column indicate activity in Table 4.

## 3. Conclusions

This method offers a simple, inexpensive, versatile, and environment friendly approach to the synthesis of a library of DHPMs. Lithium-acetate acts as an efficient promoter system of the Biginelli reaction yielding DHPMs in good-to-excellent yields. Comparative promoter efficiency of lithium-acetate and PPA to catalyze Biginelli condensation reaction is also studied under neat conditions and it has been found that PPA acts as better promoter as compared to lithium-acetate. The use of solvent-free conditions, short reaction times, excellent yields, easy workup, and compatibility with various functional groups makes the present catalytic reaction an environmentally acceptable method for the synthesis of dihydropyrimidinones and thiones.

## 4. Experimental

### 4.1. Chemical Analysis

Reagents and solvents were obtained from commercial sources and used without further purification. Melting points were determined on a Toshniwal apparatus. The spectral analyses of synthesized compounds have been carried out at SAIF, Punjab University, Chandigarh. Purity of all compounds was checked by TLC using “G-” coated glass plates and benzene: ethyl acetate (9 : 1), benzene: ethyl acetate: methanol (8.5 : 1.4 : 0.1) as eluent. IR spectra were recorded in KBr on a Perkin Elmer Infrared RXI FTIR spectrophotometer and ^1^H NMR spectra were recorded on Bruker Avance II 400 NMR Spectrometer using DMSO-d_6_ and CDCl_3_ as solvent and tetramethylsilane (TMS) as internal reference standard. The room temperature means 30–40°C. The obtained products were identified by comparison with authentic samples (synthesized by conventional process) and from their spectral (^1^H NMR and IR) data and the melting points were confirmed by comparison with those reported in the literature.

### 4.2. Experimental Procedure for the Synthesis of Dihydropyrimidinones Using Solvents (Table 1)

For comparison sake, compound 1 was synthesized by stirring at room temperature in various solvents, for example, ethanol, toluene, water, and acetonitrile for 7-8 hrs using both lithium-acetate and polyphosphoric acid as catalysts. A mixture of an aromatic aldehyde (1 mmol), *β*-dicarbonyl compound (1 mmol), urea/thiourea (2 mmol), PPA/lithium-acetate (0.5 mmol), and solvent (5 mL) was mixed in R.B. flask and the mixture was magnetically stirred at room temperature for the time needed to complete the reaction (as monitored by TLC). After completion, the reaction mixture was cooled to room temperature and poured onto crushed ice, filtered, and recrystallized by using either ethanol or ethyl acetate and pet ether (1 : 1) to afford pure product. Results are summarized in Table 1. 

### 4.3. General Procedure for the Synthesis of Dihydropyrimidinones

**Method I**

A mixture of an aromatic aldehyde (1 mmol), *β*-dicarbonyl compound (1 mmol), urea/thiourea (2 mmol) PPA/lithium-acetate (0.5 mmol) was mixed in R.B. flask and the mixture was magnetically stirred at 70–80°C for the time needed to complete the reaction (as monitored by TLC). The initial syrupy reaction mixture solidifies within 30–35 minutes. After completion, the reaction mixture was cooled to room temperature and poured onto crushed ice, filtered, and recrystallized by using either ethanol or ethyl acetate and pet ether (1 : 1) to afford pure product. The obtained products were identified from their spectral (^1^H NMR and IR) data and their literature melting points.

**Method II**

A mixture of an aromatic aldehyde (1 mmol), *β*-dicarbonyl compound (1 mmol), urea/thiourea (2 mmol), and PPA/lithium-acetate (0.5 mmol) was ground together for 5–10 min. using a mortar and pestle of appropriate size. The initial syrupy reaction mixture solidifies within 15–20 minutes. The solid mass was left overnight, then washed with cold water, and purified either by recrystallization from ethyl acetate and pet ether (1 : 1) or by column chromatography of resulting crude material over silica gel using ethyl acetate and pet ether (1.5 : 8.5) as the mobile phase. All the compounds given in Table 2 are synthesized by both the methods and it has been observed that yields of the synthesized compounds obtained by Method I is 10–15% better than that obtained by Method II.

Spectral data of newly synthesized compounds are given below.

**Compound 13 Ethyl-6-methyl-2-thioxo-4-(3-methoxyphenyl)-1,2,3,4-tetrahydropyrimidin-5-carboxylate**

IR (KBr): 3235, 3120, 2930, 2823, 1672, 1573, 1467, 1282, 1190, 1125, 723, 692 cm^−1^; ^1^H NMR (DMSO-d_6_): 1.12 (t, 3H, OCH_2_CH_3_), 2.26 (s, 3H, 6-CH_3_), 3.77 (s, 3H, OCH_3_), 4.01 (q, 2H, OCH_2_CH_3_), 5.12 (s, 1H, CH), 6.58–7.03 (m, 4H, aromatic), 7.63 (s, 1H, N–H), 9.45 (s, 1H, N–H) ppm. Analytical Calculation for C_15_H_18_N_2_O_3_S: C, 58.80; H, 5.92; N, 9.14. Found: C, 58.62; H, 5.90; N, 9.17.

**Compound 14 Ethyl-6-methyl-2-oxo-4-(2,4-dimethylphenyl)-1,2,3,4-tetrahydropyrimidin-5-carboxylate**

IR (KBr): 3234, 3110, 2933, 2833, 1703, 1649, 1511, 1455, 1276, 1175, 791 cm^−1^; ^1^H NMR (DMSO-d_6_): 1.12 (t, 3H, OCH_2_CH_3_), 2.27 (s, 3H, 6-CH_3_), 2.35 (s, 3H, 2-CH_3_), 2.35 (s, 3H, 4-CH_3_), 4.02 (q, 2H, OCH_2_CH_3_), 5.11 (s, 1H, CH), 6.74–6.82 (m, 3H, aromatic), 7.61 (s, 1H, N–H), 9.47 (s, 1H, N–H) ppm. Analytical Calculation for C_16_H_20_N_2_O_3_: C, 66.65; H, 6.99; N, 9.72. Found: C, 66.46; H, 6.97; N, 9.75.

**Compound 15 Ethyl-6-methyl-2-thioxo-4-(3,4-dimethylphen-yl)-1,2,3,4-tetrahydropyrimidin-5-carboxylate**

IR (KBr): 3313, 3171, 2989, 2842, 1668, 1575, 1460, 1373, 1332, 1259, 1192, 1118, 769 cm^−1^; ^1^H NMR (DMSO-d_6_): 1.11 (t, 3H, OCH_2_CH_3_), 2.28 (s, 3H, 6-CH_3_), 2.32 (s, 3H, 3-CH_3_), 2.33 (s, 3H, 4-CH_3_), 4.05 (q, 2H, OCH_2_CH_3_), 5.10 (s, 1H, CH), 6.72–6.81 (m, 3H, aromatic), 7.64 (s, 1H, N–H), 9.43 (s, 1H, N–H) ppm. Analytical Calculation for C_16_H_20_N_2_O_2_S: C, 63.13; H, 6.62; N, 9.20. Found: C, 63.24; H, 6.64; N, 9.17.

**Compound 19 Methyl-6-methyl-2-thioxo-4-(3,4,5-trimethoxyphenyl)-1,2,3,4-tetrahydropyrimidine-5-carboxylate**

IR (KBr): 3213, 3172, 3110, 2882, 1660, 1570, 1511, 1456, 1372, 1320, 1250, 1177, 1115, 765 cm^−1^; ^1^H NMR (DMSO-d_6_): 2.36 (s, 3H, OCH_3_), 2.13 (s, 3H, 6-CH_3_), 3.81–4.22 (s, 9H, Ar-OCH_3_), 5.12 (s, 1H, CH), 6.11–6.23 (d, 2H, aromatic), 7.76 (s, 1H, N–H), 9.42 (s, 1H, N–H) ppm. Analytical Calculation for C_16_H_20_N_2_O_5_S: C, 54.53; H, 5.72; N, 7.95. Found: C, 54.42; H, 5.71; N, 7.98.

**Compound 20 Methyl 6-methyl-2-oxo-4-(3,4,5-trimethoxyphenyl)-1,2,3,4-tetrahydropyrimidine-5-carboxylate**

IR (KBr): 3215, 3170, 3120, 2880, 1702, 1670, 1578, 1511, 1465, 1320, 1280, 1177, 1030, 762 cm^−1^; ^1^H NMR (DMSO-d_6_): 2.32 (s, 3H, OCH_3_), 2.11 (s, 3H, 6-CH_3_), 3.67–4.32 (s, 9H, Ar-OCH_3_), 5.13 (s, 1H, CH), 6.12–6.25 (d, 2H, aromatic), 7.82 (s, 1H, N–H), 9.43 (s, 1H, N–H) ppm. Analytical Calculation for C_16_H_20_N_2_O_6_: C, 57.14; H, 5.99; N, 8.33 Found: C, 57.34; H, 5.97; N, 8.35.

**Compound 22 Ethyl 6-methyl-2-oxo-4-(4-(trifluoromethyl)phenyl)-1,2,3,4-tetrahydropyrimidine-5-carboxylate**

IR (KBr): 3244, 3122, 3109, 1720, 1690, 1571, 1460, 1312, 1270, 1077, 783 cm^−1^; ^1^H NMR (DMSO-d_6_): 1.10 (t, 3H, OCH_2_CH_3_), 2.23 (s, 3H, 6-CH_3_), 3.99 (q, 2H, OCH_2_CH_3_), 5.09 (s, 1H, CH), 6.18–6.27 (m, 4H, aromatic), 7.60 (s, 1H, N–H), 9.39 (s, 1H, N–H) ppm. Analytical Calculation for C_15_H_15_F_3_N_2_O_3_: C, 54.88; H, 4.61; N, 8.53. Found: C, 54.68; H, 4.59; N, 8.56.

**Compound 23 Ethyl 6-methyl-2-oxo-4-(2-(trifluoromethyl)phenyl)-1,2,3,4-tetrahydropyrimidine-5-carboxylate**

IR (KBr): 3240, 3124, 3103, 1718, 1683, 1572, 1466, 1309, 1266, 1037, 769 cm^−1^; ^1^H NMR (DMSO-d_6_): 1.10 (t, 3H, OCH_2_CH_3_), 2.21 (s, 3H, 6-CH_3_), 3.98 (q, 2H, OCH_2_CH_3_), 5.02 (s, 1H, CH), 6.17–6.25 (m, 4H, aromatic), 7.62 (s, 1H, N–H), 9.37 (s, 1H, N–H) ppm. Analytical Calculation for C_15_H_15_F_3_N_2_O_3_: C, 54.88; H, 4.61; N, 8.53. Found: C, 54.70; H, 4.59; N, 8.55.

**Compound 24 1-Acetyl-6-(3-hydroxy-4-methoxyphenyl)-4-methyl-2-thioxo-1,2,3,6-tetrahydropyrimidine-5-carbonyl cyanide**

IR (KBr): 3251, 3432, 2225, 1728, 1381, 1250, 1115 cm^−1^; ^1^H NMR (DMSO): 2.00 (s, 1H, NH), 2.02 (s, 3H, COCH_3_), 2.27 (s, 3H, 4-CH_3_), 3.73 (s, 3H, Ar-OCH_3_), 5.56 (s, 1H, cyclic CH), 6.23 (s, 1H, Ar-H), 6.23–7.10 (d, 2H, Ar-H), 9.83 (s, 1H, ArOH) ppm. Analytical Calculation for C_16_H_15_N_3_O_4_S: C, 55.64; H, 4.38; N, 12.17. Found: C, 55.55; H, 4.40; N, 12.20.

**Compound 25. 1-Acetyl-6-(4-hydroxy-3-methoxyphenyl)-4-methyl-2-thioxo-1,2,3,6-tetrahydropyrimidine-5-carbonyl cyanide**

IR (KBr): 3254, 3435, 2233, 1722, 1382, 1240 cm^−1^; ^1^H NMR (DMSO): 2.20 (s, 1H, NH), 2.23 (s, 3H, COCH_3_), 2.27 (s, 3H, 4-CH_3_), 3.77 (s, 3H, OCH_3_), 5.57 (s, 1H, cyclic CH), 6.38–7.22 (m, 3H, Ar-H), 9.81 (s, 1H, ArOH) ppm. Analytical Calculation for C_16_H_15_N_3_O_4_S: C, 55.64; H, 4.38; N, 12.17. Found: C, 55.40; H, 4.36; N, 12.14.

**Compound 26 1-Acetyl-6-(4-hydroxyphenyl)-4-methyl-2-thioxo-1,2,3,6-tetrahydropyrimidine-5-carbonyl cyanide**

IR (KBr): 3252, 3433, 2235, 1725, 1383, 1251, 1121 cm^−1^; ^1^H NMR (DMSO): 2.40 (s, 1H, NH), 2.05 (s, 3H, COCH_3_), 2.27 (s, 3H, 4-CH_3_), 5.58 (s, 1H, cyclic CH), 6.23–7.10 (m, 4H, Ar-H), 9.88 (s, 1H, ArOH) ppm. Analytical Calculation for C_15_H_13_N_3_O_3_S: C, 57.13; H, 4.16; N, 13.33. Found: C, 56.95; H, 4.15; N, 13.35.

**Compound 27 1-Acetyl-6-(4-chlorophenyl)-4-methyl-2-thioxo-1,2,3,6-tetrahydropyrimidine-5-carbonyl cyanide**

IR (KBr): 3255, 2735, 2231, 1721, 1380, 1242 cm^−1^;^ 1^H NMR (DMSO): 2.27 (s, 3H, 4-CH_3_), 2.40 (s, 1H, NH), 2.21 (s, 3H, COCH_3_), 5.55 (s, 1H, cyclic CH), 6.38–7.22 (m, 4H, Ar-H) ppm. Analytical Calculation for C_15_H_12_ClN_3_O_2_S: C, 53.97; H, 3.62; N, 12.59. Found: C, 53.72; H, 3.64; N, 12.56.

## Figures and Tables

**Table 1:** Synthesis of Compound 1 at room temperature in the presence of PPA and Lithium-acetate in different solvents.^a^

Entry	Solvent	Catalyst	Time (hrs)	Yield (%)
1	EtOH	PPA	4	67
2	CH_3_CN	PPA	5	58
3	THF	PPA	2	73
4	Toluene	PPA	8	48
5	EtOH	Lithium-Acetate	4	63
6	CH_3_CN	Lithium-Acetate	3	73
7	THF	Lithium-Acetate	6	68
8	Toluene	Lithium-Acetate	7	43

^a^The reactions were carried out in the presence of benzaldehyde (1.0 mmol), ethyl acetoacetate (1.0 mmol), urea (2.0 mmol), and PPA or lithium-acetate (0.5 mmol) at room temperature for 6-7 hrs.

**Table 2:** Physical characterization data of compounds.^a^

Compound	R	R^1^	R^2^	X	PPA Yield (%)	LiAc Yield (%)	M.P. (°C)	
							Found	Reported
**1**	H	OEt	H	O	94	90	198–202	202-203 [25]
**2**	H	OEt	H	S	88	85	202–204	204–206 [25]
**3**	4-OCH_3_	OEt	H	O	93	91	205–207	199-200 [25]
**4**	4-OH	OEt	H	O	87	82	224–226	232–234 [25]
**5**	2-OH	OEt	H	O	85	80	195–200	201–203 [25]
**6**	3-OH	OEt	H	O	78	81	161–163	163–165 [26]
**7**	4-Cl	OEt	H	O	92	89	205–208	212-213 [25]
**8**	4-Cl	OEt	H	S	88	80	208–210	176-177 [27]
**9**	3-Cl	OEt	H	O	74	72	185–187	190–192 [12]
**10**	2-Cl	OEt	H	O	78	77	190–192	220–222 [26]
**11**	2-Cl	OMe	H	O	80	82	245–248	252-253 [28]
**12**	3-OCH_3_	OEt	H	O	92	87	210–212	220-221 [29]
**13**	3-OCH_3_	OEt	H	S	88	84	214–216	—
**14**	2,4-Dimethyl	OEt	H	O	82	85	200–202	—
**15**	3,4-dimethyl	OEt	H	S	77	78	203–205	—
**16**	3-OH, 4-OCH_3_	OEt	H	O	84	81	225–227	230–232 [30]
**17**	3-OCH_3_, 4-OH	OEt	H	O	88	83	230–232	233–235 [30]
**18**	3,4,5- Trimethoxy	OEt	H	O	92	88	202–205	216–218 [24]
**19**	3,4,5- Trimethoxy	OMe	H	S	84	78	187–190	—
**20**	3,4,5- Trimethoxy	OMe	H	O	87	84	190–192	—
**21**	4-F	OEt	H	O	91	80	189–192	192–194 [24]
**22**	4-CF_3_	OEt	H	O	88	83	165–167	—
**23**	2-CF_3_	OEt	H	O	90	87	174–176	—
**24**	3-OH, 4-OCH_3_	CN	COCH_3_	S	89	88	210–212	—
**25**	3-OCH_3_, 4-OH	CN	COCH_3_	S	84	87	190–195	—
**26**	4-OH	CN	COCH_3_	S	92	88	186–190	—
**27**	4-Cl	CN	COCH_3_	S	90	87	196–198	—

^a^The reactions were carried out by grinding aromatic aldehyde (1.0 mmol), 1,3-dicarbonyl compounds (1.0 mmol), urea/thiourea/acetyl thiourea (2.0 mmol), and PPA or lithium-acetate (0.5 mmol) in mortar and pestle at room temperature for 5–10 minutes (Method II).

**Table:** (a) Invitro cytotoxic effects of DHPMs against human lung cancer cell line A549.

		Human lung cancer cell line A549															
		% Control growth															
		Molar drug concentrations															
Codes	Compound	Experiment 1				Experiment 2				Experiment 3				Average Values			
		10^−7 ^M	10^−6 ^M	10^−5 ^M	10^−4 ^M	10^−7 ^M	10^−6 ^M	10^−5 ^M	10^−4 ^M	10^−7 ^M	10^−6 ^M	10^−5 ^M	10^−4 ^M	10^−7 ^M	10^−6 ^M	10^−5 ^M	10^−4 ^M
B-06	**06**	98.2	95.8	91.7	71.6	99.1	91.6	90.0	58.0	100.6	95.2	89.2	66.8	99.3	94.2	90.3	65.5
B-07	**16**	100.0	100.0	100.0	72.6	100.0	100.0	97.0	71.6	100.0	100.0	97.4	75.0	100.0	100.0	98.1	73.1
B-08	**18**	100.0	100.0	99.6	74.8	100.0	97.2	97.3	69.6	100.0	99.4	97.7	67.6	100.0	98.9	98.2	70.7
B-09	**23**	100.0	100.0	100.0	63.3	100.0	93.9	93.1	62.2	96.3	94.8	83.5	63.4	98.8	96.2	92.2	62.9
B-10	**24**	97.3	96.7	95.3	68.5	100.0	96.4	86.5	64.3	100.0	100.0	94.8	71.5	99.1	97.7	92.2	68.1
ADR	ADR	18.5	7.6	4.5	3.8	32.4	6.5	8.5	−10.2	24.8	15.8	10.6	0.1	25.2	10.0	7.9	−2.1

**Table:** (b)

Compound	A549		
	LC50	TGI	GI50*
**06**	>100	>100	>100
**16**	>100	>100	>100
**18**	>100	>100	>100
**23**	>100	>100	>100
**24**	>100	>100	>100
ADR	>100	85.94	*<0.1*

*GI50 ≤1 *μ*Molar is considered to be active.

**Table:** (a) Invitro cytotoxic effects of DHPMs against human breast cancer cell line MCF7.

		Human breast cancer cell line MCF7															
		% Control growth															
		Molar drug concentrations															
Codes	Compound	Experiment 1				Experiment 2				Experiment 3				Average Values			
		10^−7 ^M	10^−6 ^M	10^−5 ^M	10^−4 ^M	10^−7 ^M	10^−6 ^M	10^−5 ^M	10^−4 ^M	10^−7 ^M	10^−6 ^M	10^−5 ^M	10^−4 ^M	10^−7 ^M	10^−6 ^M	10^−5 ^M	10^−4 ^M
B-01	**02**	100.0	89.2	62.2	34.6	100.0	94.4	71.8	30.1	100.0	92.4	70.3	40.3	100.0	92.0	68.1	35.0
B-02	**03**	100.0	97.6	83.4	70.6	100.0	95.9	93.4	84.1	100.0	96.5	94.6	83.0	100.0	96.7	90.5	79.2
B-03	**07**	97.5	71.7	31.7	−14.0	100.0	85.2	38.6	−10.0	77.3	74.4	44.1	8.5	91.6	77.1	38.2	−5.2
B-04	**12**	100.0	99.2	88.5	79.8	100.0	99.8	89.3	86.7	100.0	100.0	96.2	90.3	100.0	99.7	91.3	85.6
B-05	**22**	100.0	100.0	89.3	68.9	100.0	99.1	91.1	77.0	100.0	100.0	94.9	80.1	100.0	99.7	91.8	75.3
ADR	ADR	−15.3	−43.9	−65.8	−65.0	−5.0	−33.0	−57.4	−61.6	−14.9	−33.4	−55.7	−57.3	−11.7	−36.8	−59.6	−61.3

**Table:** (b)

Compound	MCF7		
	LC50	TGI	GI50*
**02**	>100	>100	71.4
**03**	>100	>100	>100
**07**	>100	91.3	29.3
**12**	>100	>100	81.3
**22**	>100	>100	>100
ADR	54.8	<0.1	*<0.1*

*GI50 ≤1 *μ*Molar is considered to be active.
